# Investigation of Dietary Habits, Anthropometric Profile, and Hormone Levels of Women of Childbearing Age With Polycystic Ovary Syndrome (PCOS)

**DOI:** 10.7759/cureus.99967

**Published:** 2025-12-23

**Authors:** Atika Ashraf, Hira Ashraf

**Affiliations:** 1 Public Health, Health Services Academy, Islamabad, PAK; 2 Obstetrics and Gynecology, North Manchester General Hospital, Manchester, GBR

**Keywords:** anthropometric profile, dietary habits, hormone levels, lifestyle behaviors, polycystic ovary syndrome (pcos)

## Abstract

Background

Polycystic ovary syndrome (PCOS) is a common endocrine disorder characterized by metabolic, reproductive, and hormonal abnormalities. Dietary pattern, body composition, and hormonal imbalance play key roles in its presentation; however, data from South Asian populations remain limited. This study aimed to assess dietary habits, anthropometric characteristics, and hormone levels among women of reproductive age with PCOS.

Methods

A descriptive cross-sectional study was conducted among 100 women diagnosed with PCOS attending outpatient clinics at tertiary-care hospitals in Rawalpindi, Pakistan. Sociodemographic information, dietary intake, and lifestyle behaviors were recorded using a structured questionnaire. Anthropometric measurements included body mass index (BMI), waist circumference, and waist-to-hip ratio (WHR). Serum luteinizing hormone (LH), follicle-stimulating hormone (FSH), and total/free testosterone levels were analyzed. Descriptive statistics, Pearson correlations, and one-way ANOVA were performed using SPSS (IBM Corp., Armonk, USA).

Results

Participants had a mean age of 30.8 ± 5.3 years and a mean BMI of 30.7 ± 3.7 kg/m². Central adiposity was prevalent, with a mean waist circumference of 87.45 ± 3.62 cm and a mean WHR of 0.78 ± 0.12. Painful menstruation (56%), acne (52%), weight gain (52%), and difficulty conceiving (45%) were frequently reported. Dietary intake patterns showed low consumption of milk and yogurt, high intake of oils and processed foods, and suboptimal hydration levels. Mean hormonal values were 7.98 ± 1.11 IU/L (LH), 3.35 ± 0.66 IU/L (FSH), and 3.07 ± 0.63 ng/dL (testosterone). No significant correlations were observed between anthropometric measures and hormone levels. BMI did not differ significantly across physical activity categories (p = 0.645).

Conclusion

Women with PCOS in this cohort exhibited high rates of overweight and central adiposity alongside unhealthy dietary patterns and common clinical symptoms. Although anthropometric and hormonal parameters were not significantly associated, the findings underscore the need for lifestyle-focused interventions targeting nutrition and physical activity in PCOS management. Further research, including metabolic biomarkers and longitudinal designs, is recommended to better understand these relationships in South Asian populations.

## Introduction

Polycystic ovary syndrome (PCOS) is one of the most common endocrine and metabolic disorders affecting women of reproductive age, characterized by hyperandrogenism, ovulatory dysfunction, and polycystic ovarian morphology [1]. It affects an estimated 6-13% of women worldwide, though prevalence varies by diagnostic criteria and population studied [2]. According to studies, around 65-70 million women globally are living with PCOS, making it a major public health concern, particularly in low- and middle-income countries [3]. In addition to its reproductive manifestations-irregular menses, infertility, acne, and hirsutism-PCOS is also associated with long-term metabolic risks, including insulin resistance, obesity, dyslipidemia, type 2 diabetes mellitus, and cardiovascular disease [4,5].

Recent international evidence‑based guidelines emphasize a standardized approach to the diagnosis and management of PCOS, aimed at improving consistency in clinical practice and outcomes. In adults, PCOS is diagnosed when at least two of the following three features are present: clinical or biochemical hyperandrogenism, ovulatory dysfunction, and polycystic ovarian morphology on ultrasound or elevated anti‑Müllerian hormone (AMH) levels, with exclusion of other etiologies. Importantly, irregular menstrual cycles and hyperandrogenism alone may suffice for diagnosis without imaging in adults, while AMH can be considered an alternative to ultrasound when appropriate. In adolescents, both hyperandrogenism and ovulatory dysfunction must be present for diagnosis, and imaging is generally not recommended due to low specificity in this age group. The guidelines also recognize a broader spectrum of associated features, including metabolic risk factors, psychological comorbidities, and long‑term cardiometabolic outcomes, and reinforce the central role of lifestyle interventions and patient‑centered care in management [6].

Dietary intake plays a central role in the pathophysiology and management of PCOS. Poor dietary quality, high glycemic load, excessive caloric intake, and frequent consumption of processed foods have been linked with weight gain, insulin resistance, and worsening hyperandrogenism in women with PCOS [7,8]. Lifestyle modification, including improved diet quality and physical activity, is widely recognized as the first-line therapeutic approach, with evidence showing clinically meaningful benefits on metabolic parameters, menstrual regularity, and ovulatory function [9,10]. However, dietary patterns of women with PCOS vary widely by cultural and socioeconomic context, and little is known about the habitual dietary practices of Pakistani women affected by the disorder.

Anthropometric indicators such as body mass index (BMI), waist circumference, and waist-to-hip ratio (WHR) are strong predictors of metabolic dysfunction in PCOS. South Asian women exhibit higher visceral adiposity even at lower BMI levels, increasing their risk of insulin resistance and cardiometabolic disease [11,12]. Given that more than half of women with PCOS are overweight or obese, evaluating anthropometric characteristics is essential to understanding disease burden and progression in this population [13].

Hormonal imbalances remain the cornerstone of PCOS diagnosis and include elevated luteinizing hormone (LH), altered LH/FSH (follicle-stimulating hormone) ratios, and increased total or free testosterone levels. These hormonal alterations contribute to anovulation, menstrual irregularities, and clinical hyperandrogenism [14]. Variations in hormonal patterns may also reflect underlying metabolic status and lifestyle influences, emphasizing the need to evaluate biochemical markers alongside dietary and anthropometric factors.

While numerous studies have assessed either metabolic complications or reproductive outcomes in PCOS, limited evidence examines the combined relationship between dietary habits, anthropometric markers, and hormonal profiles in Pakistani women of reproductive age. This gap is particularly relevant given the increasing burden of obesity, sedentary lifestyles, and dietary transitions in the region. Understanding these interlinked domains is critical to improving lifestyle-based interventions and optimizing clinical management. Therefore, this study aimed to investigate the dietary habits, anthropometric profile, and hormone levels of women of childbearing age with PCOS attending tertiary care hospitals in Rawalpindi, Pakistan. It also aimed to examine potential associations between dietary patterns, anthropometric measures, and hormone levels, as well as providing region-specific insights into lifestyle and metabolic factors that may inform clinical management and public health strategies.

## Materials and methods

Study design and setting

This observational, descriptive cross-sectional study was conducted from June to August 2023 in the outpatient departments of three tertiary-care hospitals located in Rawalpindi, Pakistan. These hospitals collectively manage approximately 150,000-200,000 outpatient visits annually, providing specialized care across gynecology, endocrinology, and metabolic disorders, including reproductive health services. Ethical approval for the study was obtained from the Internal Review Committee of the Health Services Academy, Islamabad, under approval number 000211/HSA/MSPH-2021, dated 1st June, 2023. All eligible participants provided written informed consent after receiving an explanation of the study purpose and procedures. Participant confidentiality was ensured by assigning unique identification codes in place of personal information. The study was conducted in accordance with the ethical principles outlined in the Declaration of Helsinki.

Study population

The study population included women aged 18 to 40 years with a confirmed diagnosis of PCOS based on the Rotterdam criteria, requiring the presence of at least two of the following: oligo- or anovulation, clinical or biochemical hyperandrogenism, and polycystic ovarian morphology on ultrasound. On average, 70-80 women with PCOS visit the participating hospitals monthly, providing an adequate sampling pool.

Women with conditions that could confound the diagnosis or evaluation of PCOS were excluded. This included ovarian or endocrine neoplasms, autoimmune disorders, pregnancy, and breastfeeding. Additionally, women with menstrual disorders unrelated to PCOS were excluded. Abnormal bleeding unrelated to PCOS was assessed using a detailed clinical history and laboratory evaluation. Participants reporting heavy menstrual bleeding, intermenstrual bleeding, or irregular cycles not consistent with oligo/anovulation were evaluated for alternative causes, including thyroid dysfunction, coagulation disorders, and structural uterine pathologies (assessed via ultrasound and laboratory tests). Only women who met the PCOS diagnostic criteria and provided written informed consent were enrolled.

Sampling technique and sample size

A sample size of 100 participants was determined using a prevalence estimate of 52% for PCOS in Pakistani women of reproductive age, with a 95% confidence level and a 5% margin of error. This estimate is supported by recent studies reporting PCOS prevalence of 52% among similar clinical populations in Pakistan [15]. To account for potential non-response, the sample size was increased to 100. A convenience sampling method was employed due to the outpatient clinical flow and feasibility considerations.

Data collection tool

Data were collected using a structured, pretested questionnaire administered through face-to-face interviews. The tool comprised of sociodemographic information such as age, marital status, occupation, and education, anthropometric measurements including height, weight, BMI, waist circumference, hip circumference, and WHR, hormonal profile based on the most recent laboratory results for LH, FSH, and total/free testosterone, and dietary intake assessed through a food frequency questionnaire (FFQ) adapted from validated instruments and tailored to local dietary patterns. The questionnaire included commonly consumed foods in the region, such as wheat-based staples (roti, paratha), rice, lentils (daal), vegetables, fruits, dairy products, meat and poultry, fried foods, sweets, sugar-sweetened beverages, and fast foods. Portion size and frequency of consumption were recorded to reflect the typical dietary habits of women in the local setting. Difficulty conceiving was assessed through self-report. Participants were asked whether they had ever attempted to conceive for at least 12 months without success. Women who had never attempted conception, including those who were unmarried or not planning pregnancy, were categorized as “not applicable” and were not considered as having difficulty conceiving.

Anthropometric and hormonal assessment

All anthropometric measurements were obtained using standardized techniques in the outpatient departments of the participating tertiary-care hospitals. Measurements were taken in a designated clinical area to ensure privacy. Height was measured to the nearest 0.1 cm using a stadiometer, with participants standing barefoot in an upright position. Weight was measured to the nearest 0.1 kg using a calibrated digital scale, with participants wearing light clothing. BMI was calculated as weight (kg) divided by height in meters squared (m²). Waist circumference was measured at the midpoint between the lowest rib and the iliac crest, and hip circumference at the widest portion of the hips, using a non-stretchable measuring tape. WHR was subsequently computed. All anthropometric measurements were performed by trained data collectors following standardized protocols to ensure consistency.

Hormonal profiles were obtained from participants’ most recent laboratory reports conducted as part of routine clinical care. The tests were typically performed during the early follicular phase (days 2-5) of the menstrual cycle, as indicated in the lab reports. As the investigators did not perform the blood draws or assays themselves, detailed specifications of the test kits were not available; results were used as reported by the certified hospital laboratories.

Physical activity assessment

Physical activity was assessed using self-reported frequency questions included in the study questionnaire. Participants were asked how often they engaged in physical activity, including walking, exercise, or routine physical exertion, and responses were categorized as daily, three to four times per week, one to two times per week, or never. No standardized or validated physical activity questionnaire was used.

Pilot testing and data quality assurance

The structured questionnaire was designed by the study investigators specifically for this research to capture sociodemographic information, anthropometric measurements, hormonal profiles (from lab reports), and habitual dietary patterns relevant to the local population.

The questionnaire was piloted among 10-15 women with PCOS in a non-study setting to ensure clarity, cultural appropriateness, and suitability. Feedback from the pilot was incorporated to refine question wording, response options, and flow. Data collectors were trained on interview techniques, anthropometric measurement procedures, and ethical considerations to ensure consistency and reliability. While formal psychometric validation was not performed, the pilot testing and standardized data collection procedures provided confidence in the tool’s clarity and reliability for the study objectives.

Data analysis

Data were entered and analyzed using IBM SPSS Statistics version 26 (IBM Corp., Armonk, USA). Descriptive statistics, including means, standard deviations (SD), frequencies, and percentages, were used to summarize sociodemographic characteristics, anthropometric measures, hormone levels, and dietary patterns. Chi-square tests were applied to assess associations between categorical variables. Pearson correlation coefficients were calculated to evaluate relationships between dietary intake, anthropometric indicators, and hormonal parameters. A p-value of <0.05 was considered statistically significant.

## Results

The sociodemographic and clinical characteristics of the participants are summarized in Table 1. Overall, nearly half of the participants were married, and the majority were literate. Students constituted the largest occupational group. Most women reported experiencing PCOS-related symptoms for one to five years, and approximately half were currently receiving treatment. Regarding clinical features, painful menstruation, weight gain, and acne were the most commonly reported symptoms, while a smaller proportion reported irregular or delayed menstruation and abnormal facial hair growth.

**Table 1:** Sociodemographic and Clinical Characteristics of Participants (n = 100) PCOS, polycystic ovary syndrome

Variable	Category	n (%)
Marital status	Single	47 (47.0)
	Married	48 (48.0)
	Divorced	5 (5.0)
Education	Literate	88 (88.0)
	Illiterate	12 (12.0)
Occupation	Student	38 (38.0)
	Housewife	32 (32.0)
	Employed	30 (30.0)
PCOS symptoms duration	<1 year	24 (24.0)
	1–5 years	55 (55.0)
	>5 years	21 (21.0)
Currently receiving treatment	Yes	53 (53.0)
	No	47 (47.0)
Key clinical features	Painful menstruation	56 (56.0)
	Irregular/delayed menstruation	33 (33.0)
	Weight gain	52 (52.0)
	Acne	52 (52.0)

Anthropometric and hormonal characteristics

The mean age of participants was 30.8 ± 5.3 years. The average BMI was 30.7 ± 3.7 kg/m². Mean waist circumference was 87.45 ± 3.62 cm, and mean WHR was 0.78 ± 0.12. Hormonal analysis showed a mean LH level of 7.98 ± 1.11 IU/L, a mean FSH level of 3.35 ± 0.66 IU/L, and a mean total/free testosterone level of 3.07 ± 0.63 ng/dL (Table 2).

**Table 2:** Anthropometric and Hormonal Characteristics of Participants BMI, body mass index; WHR, waist-to-hip ratio; LH, luteinizing hormone; FSH, follicle stimulating hormone

Variable	Mean ± SD
Age (years)	30.8 ± 5.3
BMI (kg/m²)	30.7 ± 3.7
Waist circumference (cm)	87.45 ± 3.62
WHR	0.78 ± 0.12
LH (IU/L)	7.98 ± 1.11
FSH (IU/L)	3.35 ± 0.66
Testosterone (ng/dL)	3.07 ± 0.63

Dietary intake and lifestyle patterns

Daily water intake varied, with 33% consuming six to eight cups per day, 24% consuming three to five cups, 22% consuming more than eight cups, and 21% consuming fewer than three cups daily. Sugar cravings were reported by 42% of the sample. Daily consumption of milk was reported by 24% of participants, while 30% consumed milk three to four times per week, and 28% reported never drinking milk. Only 12% consumed yogurt daily, though 55% consumed it three to four times per week. Eggs were consumed daily by 32% of participants, with 57% consuming them three to four times per week. Potatoes were the most frequently consumed vegetable, with 84% reporting daily intake. Consumption of fats was widespread; 67% reported daily use of cooking oil, and 64% reported daily use of banaspati ghee. Processed foods were also common, with daily intake of French fries (32%), chips (25%), and popcorn (30%). Physical activity patterns showed that only 23% of participants exercised daily, while 25% exercised one to two times per week, 24% exercised three to four times per week, and 28% never exercised (Table 3).

**Table 3:** Dietary Intake and Lifestyle Patterns of Participants (n = 100)

Variable	Category	n (%)
Daily water intake	<3 cups	21 (21.0)
	3–5 cups	24 (24.0)
	6–8 cups	33 (33.0)
	>8 cups	22 (22.0)
Sugar cravings	Yes	42 (42.0)
Milk intake	Daily	24 (24.0)
	3–4 times/week	30 (30.0)
	Every 2–3 weeks	18 (18.0)
	Never	28 (28.0)
Yogurt intake	Daily	12 (12.0)
	3–4 times/week	55 (55.0)
	Every 2–3 weeks	13 (13.0)
	Never	20 (20.0)
Egg intake	Daily	32 (32.0)
	3–4 times/week	57 (57.0)
	Every 2–3 weeks	5 (5.0)
	Never	6 (6.0)
Potato intake	Daily	84 (84.0)
	3–4 times/week	11 (11.0)
	Never	5 (5.0)
Daily cooking oil use	Yes	67 (67.0)
Daily banaspati ghee use	Yes	64 (64.0)
Daily French fries consumption	Yes	32 (32.0)
Daily chips consumption	Yes	25 (25.0)
Daily popcorn consumption	Yes	30 (30.0)
Physical activity frequency	Daily	23 (23.0)
	1–2 times/week	25 (25.0)
	3–4 times/week	24 (24.0)
	Never	28 (28.0)

Correlation between anthropometric and hormonal parameters

Pearson correlation analysis revealed no statistically significant associations between BMI and LH (r = -0.085, p = 0.402), FSH (r = -0.068, p = 0.499), or testosterone levels (r = -0.027, p = 0.790). Similarly, waist circumference showed no significant correlation with LH (r = 0.030, p = 0.769), FSH (r = -0.002, p = 0.982), or testosterone (r = 0.095, p = 0.346).

WHR demonstrated a weak negative correlation with testosterone (r = -0.196), which approached but did not reach statistical significance (p = 0.051). Marital status was not significantly correlated with any anthropometric or hormonal variable (Table 4).

**Table 4:** Pearson Correlation Coefficients between Anthropometric and Hormonal Parameters Values represent Pearson correlation coefficients (r) and corresponding p-values evaluating associations between anthropometric indicators and hormone levels. p-value <0.05 was considered significant. BMI, body mass index; WHR, waist-to-hip ratio; LH, luteinizing hormone; FSH, follicle stimulating hormone

Anthropometric Variable	Hormonal Variable	Correlation (r)	p-value
BMI (kg/m²)	LH (IU/L)	–0.085	0.402
	FSH (IU/L)	–0.068	0.499
	Testosterone (ng/dL)	–0.027	0.79
Waist circumference (cm)	LH (IU/L)	0.03	0.769
	FSH (IU/L)	–0.002	0.982
	Testosterone (ng/dL)	0.095	0.346
Waist-to-hip ratio (WHR)	LH (IU/L)	0.018	0.859
	FSH (IU/L)	–0.053	0.598
	Testosterone (ng/dL)	–0.196	0.051
Marital status	BMI (kg/m²)	0.082	0.419
	Waist circumference (cm)	–0.065	0.522
	WHR	0.14	0.166
	LH (IU/L)	–0.090	0.375
	FSH (IU/L)	0.113	0.262
	Testosterone (ng/dL)	–0.068	0.503

Association between BMI and physical activity

A one-way ANOVA was conducted to compare BMI across four physical activity categories. The assumption of homogeneity of variances was met (Levene’s test p = 0.325). Mean BMI ranged from 29.87 ± 4.42 kg/m² among those exercising daily to 31.15 ± 3.33 kg/m² among those who never exercised. However, these differences were not statistically significant (F(3, 96) = 0.556, p = 0.645) (Table 5).

**Table 5:** Comparison of BMI across Physical Activity Categories One-way ANOVA with Levene’s test for homogeneity was used to evaluate mean BMI differences among physical activity groups.

Physical Activity Frequency	n	BMI (Mean ± SD)	F-value	p-value
Daily	23	29.87 ± 4.42		
1–2 times/week	25	30.98 ± 3.61		
3–4 times/week	24	30.65 ± 3.68		
Never	28	31.15 ± 3.33		
ANOVA Results	—	—	0.556	0.645

Association between categorical variables

Chi-square analyses were performed to explore associations between selected categorical variables. There was no significant association between daily banaspati ghee consumption and BMI category (χ² = 0.60, p = 0.44). Similarly, irregular or delayed menstruation was not significantly associated with difficulty conceiving, with comparable proportions of participants reporting fertility issues in both regular and irregular cycle groups (χ² = 0.004, p = 0.95) (Table 6). These findings indicate that neither dietary fat intake nor menstrual irregularity was related to obesity status or self-reported fertility difficulty in this cohort.

**Table 6:** Chi-Square Test Results for Associations between Categorical Variables Pearson chi-square tests were used to evaluate associations between categorical variables. P-value <0.05 was considered significant.

Association Tested	χ²	p-value
Daily banaspati ghee use × BMI category	0.596	0.44
Irregular or delayed menstruation × Difficulty conceiving	0.004	0.95

## Discussion

This study investigated the dietary habits, anthropometric characteristics, and hormone levels of women of reproductive age diagnosed with PCOS. The findings show that participants exhibited elevated BMI and central adiposity, along with varied dietary patterns characterized by low dairy consumption and frequent intake of high-fat and processed foods. Although clinical symptoms such as painful menstruation, acne, and weight gain were prevalent, no significant correlations were found between anthropometric parameters and hormone levels. Additionally, BMI did not differ significantly across categories of physical activity. These results offer valuable insights into the lifestyle and metabolic profile of PCOS women in this region.

Our findings are consistent with previous literature indicating unhealthy dietary patterns among women with PCOS. Lim et al. [16] reported that women with PCOS consume more calorie-dense foods, refined carbohydrates, and fewer nutrient-rich foods compared to healthy controls. Similarly, Manta et al. [17] observed that PCOS is commonly associated with higher intake of high-glycemic and processed foods, aligning with our participants’ frequent consumption of fries, chips, cooking oil, and banaspati ghee. Studies from South Asian populations, such as Hajivandi et al. [18], have also reported low dairy intake and irregular dietary habits among women with PCOS, which is consistent with our findings. Hydration patterns in our sample, where a substantial proportion consumed less than six cups of water daily, also reflect global observations linking poor hydration and dietary quality with PCOS, as discussed by Barrea et al. [19] and Nowosad et al. [20].

The anthropometric trends observed in this study likewise align with existing data. Increased central adiposity is a well-documented feature of PCOS, even among individuals with similar BMI values. Escobar-Morreale [13] emphasized that abdominal fat accumulation is a hallmark of PCOS across ethnicities. Our participants demonstrated elevated waist circumference and WHR, supporting this established pattern. Additionally, the variability in clinical symptoms such as acne, hirsutism, and menstrual irregularity is consistent with Azziz et al. [21], who highlighted the heterogeneity of PCOS manifestations globally [6].

Regarding hormonal findings, our mean LH, FSH, and testosterone values fall within ranges commonly reported in PCOS studies. Importantly, our study found no significant correlations between anthropometric indicators (BMI, waist circumference, WHR) and hormonal levels. This aligns with the Rotterdam consensus report, which notes that hormonal markers in PCOS demonstrate considerable variability and are not consistently associated with body composition measures [1]. Moran et al. [9] also found inconsistent relationships between BMI and androgen excess across populations, supporting the lack of correlation in our cohort.

However, several studies demonstrate contradictory findings. Luo et al. [22] reported a significant association between BMI and the LH/FSH ratio, suggesting that obesity may exacerbate hormonal dysregulation in PCOS [9]. Mansour et al. [23] found that higher BMI was strongly associated with elevated testosterone levels, a relationship not observed in our sample. Additionally, Dumesic et al. [24] reported that WHR and central adiposity are frequently associated with hyperandrogenism, whereas our WHR-testosterone correlation only approached significance (p = 0.051). Differences between our findings and prior research may stem from the relatively homogeneous BMI range in our sample, population-specific genetic or metabolic characteristics, and differences in dietary patterns or assay methodologies.

Another contrast was observed in the relationship between lifestyle factors and BMI. While our study found no significant difference in BMI across physical activity categories, it has been reported that irregular dietary patterns and low activity levels were significantly correlated with higher BMI among women with PCOS [25,26]. This discrepancy may be attributable to differences in sample size, measurement of physical activity, or lifestyle diversity in different populations.

An important consideration is the potential influence of social status and the role of patient and physician perspectives in PCOS management. Education, occupation, and marital status may affect health literacy, access to healthcare, and adherence to lifestyle or medical interventions, potentially confounding observed associations. Furthermore, women’s experiences, expectations, and beliefs, together with physicians’ clinical approaches, play a critical role in treatment adherence and follow-up. Aligning patient and physician perspectives through patient-centered communication and individualized counseling has been shown to improve satisfaction and engagement in hormonal health management [27], highlighting the importance of considering these factors in PCOS care and research.

The non-significant correlations in our study may also be explained by several factors. The homogeneity of BMI values (24-38 kg/m²) may have reduced the variability needed to detect associations. Additionally, dietary and lifestyle factors in the local population are relatively uniform, potentially masking differences. Hormone levels were measured once, without standardizing for menstrual cycle phase, which may influence hormonal variability. Finally, the cross-sectional design limits the ability to explore temporal or causal relationships.

Despite these limitations, this study has notable strengths. It is one of the few studies in Pakistan to comprehensively assess dietary intake, anthropometric measurements, and hormonal profiles simultaneously in women with PCOS. The use of standardized anthropometric methods and detailed dietary assessment contributes to the reliability of the findings. This study provides region-specific insights into PCOS in Pakistani women, which is particularly valuable given potential ethnic and cultural differences in dietary habits, lifestyle behaviors, and social determinants of health. South Asian populations, including Pakistanis, often exhibit higher central adiposity and distinct metabolic profiles at lower BMI compared to Western populations. Moreover, dietary patterns, food preferences, and social roles-such as women’s household responsibilities and access to healthcare-may differ substantially from other populations, influencing both disease manifestation and management. These region-specific characteristics, while a potential limitation for generalizability, also represent strength, as local data on PCOS are scarce and can inform tailored lifestyle interventions, culturally appropriate counseling, and public health strategies in this setting.

Future research should incorporate larger, more diverse samples and include additional markers such as insulin resistance indices, lipid profiles, and inflammatory markers. Longitudinal and interventional studies would also help clarify causal pathways and evaluate the effectiveness of dietary and lifestyle modifications in PCOS management.

## Conclusions

This study provides an overview of the dietary habits, anthropometric profile, and hormonal characteristics of women of reproductive age with PCOS in a tertiary-care setting in Pakistan. Most participants exhibited elevated BMI and central adiposity, alongside dietary patterns marked by low intake of dairy products and frequent consumption of high-fat and processed foods. Although common PCOS-related symptoms such as painful menstruation, acne, and weight gain were widely reported, no significant associations were observed between anthropometric indicators and hormonal levels, or between BMI and physical activity frequency. These findings highlight the importance of addressing lifestyle behaviors, particularly nutrition and physical activity, as part of comprehensive PCOS management in this population. While the absence of significant correlations suggests a complex interplay between metabolic and hormonal factors, the study contributes valuable region-specific data that can inform future preventive and therapeutic strategies. Larger, longitudinal studies incorporating metabolic biomarkers are recommended to further elucidate these relationships and support targeted interventions for women with PCOS.
